# Cationic Liposome- Multi-Walled Carbon Nanotubes Hybrids for Dual siPLK1 and Doxorubicin Delivery *In Vitro*

**DOI:** 10.1007/s11095-015-1707-1

**Published:** 2015-06-18

**Authors:** Sara Pereira, Jin Lee, Noelia Rubio, Hatem A. F. M. Hassan, Izzat Bin Mohamed Suffian, Julie T. W. Wang, Rebecca Klippstein, Belén Ballesteros, Wafa’ T. Al-Jamal, Khuloud T. Al-Jamal

**Affiliations:** Institute of Pharmaceutical Science, King’s College London, Franklin-Wilkins Building, 150 Stamford Street, London, SE1 9NH UK; ICN2 - Institut de Catala de Nanociencia i Nanotecnologia, Campus UAB, 08193 Bellaterra, Barcelona, Spain; School of Pharmacy, University of East Anglia, Norwich Research Park, Norwich, NR4 7TJ UK

**Keywords:** A549, cancer cells, gene silencing, multi-walled carbon nanotubes, siRNA

## Abstract

**Purpose:**

To formulate *f*-MWNTs-cationic liposome hybrids for the simultaneous delivery of siPLK1 and doxorubicin to cancer cells.

**Method:**

*f*-MWNTs-cationic liposome hybrids were prepared by the thin film hydration method where the lipid film was hydrated with 100 μg/ml or 1 mg/ml of ox-MWNTs-NH_3_^+^ or MWNTs-NH_3_^+^ in 5% dextrose. siRNA complexation and protection ability was determined by agarose gel electrophoresis. *f*-MWNTs and liposome interaction was evaluated using Nile Red (NR) fluorescence spectroscopy. Cellular uptake in A549 cells was assessed by flow cytometry. Silencing of target proteins was determined by Luciferase and MTT assays. Sub-G1 analysis was performed to evaluate apoptosis following co-delivery of siPLK1 and Doxorubicin (Dox).

**Results:**

Zeta potential and siRNA complexation profile obtained for all hybrids were comparable to those achieved with cationic liposomes. ox-MWNTs-NH_3_^+^ showed greater extent of interaction with cationic liposomes compared to MWNTs-NH_3_^+^. ox-MWNTs-NH_3_^+^ was able to protect siRNA from nuclease-mediated degradation. Enhanced cellular uptake of both the carrier and loaded siRNA in A549 cell, were observed for this hybrid compared to the liposomal carrier. A synergistic pro-apoptotic effect was obtained when siPLK1 silencing was combined with doxorubicin treatment for the hybrid:siRNA complexes compared to the lipoplexes, in A549 cells *in vitro*.

**Conclusions:**

*f*-MWNTs-cationic liposome hybrid designed in this study can serve as a potential vehicle for the co-delivery of siRNA and cytotoxic drugs to cancer cells *in vitro*.

**Electronic supplementary material:**

The online version of this article (doi:10.1007/s11095-015-1707-1) contains supplementary material, which is available to authorized users.

## Introduction

Small interfering RNA (siRNA) has emerged as a therapeutic strategy for various diseases, including cancer, due to its target-specific gene silencing. Moreover, down-regulation of genes that are overexpressed in tumors by siRNA presents advantages over plasmid DNA therapy. As siRNA acts at the cytoplasm level, it does not need to cross the nuclear envelope contrary to DNA which needs to be delivered to the cell nucleus (1). However, due to its high molecular weight and negative charge, cellular uptake and subsequent intracellular trafficking to the effector complex (RNA-induced silencing complex, RISC) are hampered and represent major technical hurdles for the efficacy of siRNA (2). Thus, in the past few years researchers have focused on the development of suitable delivery systems that would make clinical practice of siRNA therapy possible. Approaches to overcome these limitations have relied on non-viral siRNA carriers based on cationic liposomes or polymers able to spontaneously complex and condense genetic materials (3). Nevertheless, these systems are characterized by a lack of efficiency accompanied by a high level of toxicity rendering them mostly inadequate for *in vivo* applications (2).

Recently, a unique type of nonviral gene vector has emerged, based on functionalized carbon nanotubes (*f*-CNTs) (4). CNTs are ordered structures with high aspect ratio and unique features such as high mechanical strength, high thermal and electrical conductivity (5). In addition, during the last decade, their potential use in the biomedical field has also attracted great interest due to CNTs ability to translocate into the cytoplasm by directly crossing the plasma membrane *via* an endocytosis-independent route where they act like “nanoneedles” without inducing apoptosis (4–6). The main problem associated with the use of CNTs is their hydrophobicity which leads to poor dispersibility. However, this can be circumvented by surface chemical functionalization, improving CNTs dispersibility in physiological media and consequently broadening the spectrum of their potential biological applications (7–9).

In the present study, we aimed at formulating functionalized multi-walled carbon nanotubes (*f*-MWNT)-cationic liposome hybrids for the co-delivery of siRNA and a cytotoxic drug *in vitro*. Doxorubicin (Dox) was chosen as the model drug as it is commonly used in the treatment of a wide range of cancers. Polo-like kinase 1 (PLK1) is a serine/threonine kinase with key roles in cell division and checkpoint regulation of mitosis. PLK1 is overexpressed in several types of cancer and correlates with high tumor grades. siRNA-mediated silencing of PLK1 in several cancer cell lines was shown to result in decreased cell viability with induction of apoptosis, defects in several mitosis processes and G2/M phase arrest (10). Other studies showed that PLK1 down-regulation sensitizes cancer cells to chemotherapy and can revert chemoresistance (11–14).

In this study, a series of *f*-MWNT-cationic liposome hybrids were formulated and tested for their siRNA binding ability, stability, cellular uptake, *in vitro* gene silencing efficiency, induction of apoptosis and siPLK1 and Dox co-delivery efficiency. Liposomes consisted of 1,2-dioleoyl-3-trimethylammonium-propane (DOTAP) and cholesterol (DOTAP:chol, 2:1 molar ratio), one of the most widely used transfection agents. Two types of *f*-MWNTs were used: MWNT-NH_3_^+^ (−455.5 +/− 185.42 nm in length, 20–30 nm in diameter) and ox-MWNT-NH_3_^+^ (−277.5 +/− 152.93 nm nm in length, 20–30 nm in diameter). *f*-MWNT-liposome hybrids were prepared at two different *f*-MWNT:total lipid mass ratios (5.3%:94.7% and 35.8%:64.2%).

## Materials and Methods

### Materials

**Fig. 1:**
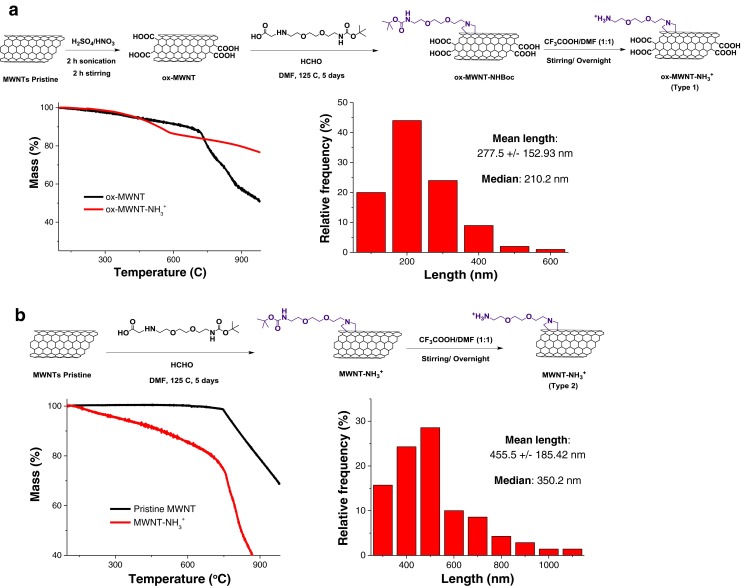
Characterization of the synthesized ox-MWNT-NH_3_^+^ (**type 1**) and MWNT-NH_3_^+^ (**type 2**) derivatives. (**a**) Scheme of the synthetic route used for the synthesis of ox-MWNT-NH_3_^+^ derivatives. TGA analysis of the ox-MWNT (*black line*) and ox-MWNT-NH_3_^+^ (*red line*). ox-MWNT showed a drop in weight loss at 150°C, due to the loss in the carboxylic groups introduced on CNT surface, and at 600°C due to graphitic-skeleton decomposition. ox-MWNT-NH_3_^+^ showed an increase in the weight loss (13% at 600°C) due to the introduction of functional groups from the cycloaddition reaction. (**b**) Scheme of the synthetic route used for the synthesis of MWNT-NH_3_^+^ derivatives. TGA analysis of the pristine MWNT (*black line*) showed its thermal stability up to 750°C. MWNT-NH_3_^+^ (*red line*) displayed an increase in the weight loss (15% at 600°C) due to the introduction of functional groups from the cycloaddition reaction. Length distribution (mean ± SD) and median, determined from TEM measurements (*n* = 100) are displayed on length distribution histograms.

Pristine MWNTs were purchased from Nanostructured and Amorphous Materials Inc. (Houston, USA); Batch: 1237YJS 95%, outer diameters between 20 and 30 nm, and lengths between 0.5 and 2 μm). ox-MWNT-NH_3_^+^ (type **1**) and MWNT-NH_3_^+^ (type **2**) (Fig. 1) were functionalized using 1,3-dipolar cycloaddition as described by Prato and co-workers (15). 1,2-dioleoyl-3-trimethylammonium-propane (chloride salt) (DOTAP, 99%) was obtained from Avanti Polar Lipid (USA). Cholesterol, chloroform, methanol, dextrose, doxorubicin hydrochloride (Dox), MTT (3-(4,5-dimethylthiazol-2-yl)-2,5-diphenyltetrazolium bromide), dimethylsulfoxide (DMSO), paraformaldehyde and Ribonuclease A from bovine pancreas RNAse (R6513-50MG) were purchased from Sigma-Aldrich (UK). 0.2 μm filter was from Millipore (UK). Dulbecco’s modified Eagle medium (DMEM), Ham’s F-12 medium, fetal bovine serum (FBS), penicillin/streptomycin, phosphate buffered saline (PBS), Lipofectamine 2000, propidium iodide (PI), and agarose were purchased from Life Technologies (Invitrogen, UK). 6× Orange Loading Dye was from Fermentas (UK) and 0.3× GelRed stain was from Biotium (USA). Luciferase assay kit was obtained from Promega Corporation (Madison, WI) and BCA protein assay kit from Thermal Scientific (UK). Noncoding siNEG and ATTO 655-labeled siNEG (antisense sequence, 5′-CAUCGUCGAUCGUAGCGCAA-3′, #4754184; sense sequence, 5′-GCGCUACGAUCGACGAUGGG-3′, #4754185), and siPlk-1 (sequence 5′-CCUUGAUGAAGAAGAUCACdTdT-3′) were purchased from Eurogentec (Belgium). The human epithelial lung carcinoma A549 (CCL-185) cell line was from American Type Culture Collection (ATCC). A549-Luc cells were purchased from Caliper (Perkin Elmer). Silencer Luciferase siRNA (siLuc) (AM4629) was obtained from Ambion (USA). All chemical substances and solvents were used without further purification.

### Chemical Functionalization of MWNTs

To prepare ammonium-functionalized oxidized multiwalled carbon nanotubes (ox-MWNTs- NH_3_^+^) **type 1**, pristine MWNTs were firstly oxidized under stirring for 2 h followed by sonication in a water bath (20 W, 40 kHz) for 2 h in a sulfuric acid/nitric acid solution (3:1 *v/v*, 98 and 65%, respectively) at room temperature. Deionized water (DI) was then carefully added and the oxidized MWNTs were filtered (Omnipore GTTP membrane filtration, 0.22 μm), The collected black solid was washed with DI (300 mL), 5% NaOH solution (300 mL) and methanol (200 mL) yielding 42 mg of ox-MWNT. ox-MWNTs-NH_3_^+^ (**type 1**) was subjected to the 1,3 dipolar cycloaddition reaction (16). In brief, ox-MWNT (20 mg) were suspended in 20 ml of dimethylformamide (DMF) and sonicated for 10 min. After sonication, the tertiary-butyloxycarbonyl (Boc)-protected amino acid (150 mg, 0.51 mmol) (Fig. 1a) and paraformaldehyde (18.5 mg, 0. 51 mmol) were added stepwise (30 mg amino acid and 3.7 mg formaldehyde every 24 h), and the mixture was heated at 125°C for 5 days. Multiple centrifugation steps (1811 g, 10 min) were carried out to separate the unreacted MWNTs (remain suspended) from the *f*-MWNTs, which were then filtered through a 0.2 μm PTFE filter and the collected black solid was washed with 100 ml of dimethylformamide (DMF) and methanol yielding 18 mg of ox-MWNT-NHBoc. For Boc deprotection, 15 mg of ox-MWNT-NHBoc were suspended in 10 ml DMF, and sonicated for 10 min. After sonication, 10 ml of trifluoroacetic acid (TFA) were added to the reaction mixture. The solution was stirred for 24 h and the *f*-MWNTs were filtered through a 0.2 μm PTFE filter. The collected black solid was washed with 10 ml of DMF and methanol yielding 13 mg of ox-MWNT-NH_3_^+^ (**type 1**) (thermogravimetric analysis (TGA) mass loss of 13%, 193 μmol functional groups/g MWNTs). ox-MWNTs-NH_3_^+^ were dispersed in 5% dextrose at concentrations of 100 μg/mL and 1 mg/mL, affording **1-L** and **1-H**, respectively.

Ammonium-functionalized multiwalled carbon nanotubes (MWNTs-NH_3_^+^, **type 2**) were prepared directly from pristine MWNTs following the 1,3 dipolar cycloaddition reaction as-described above, without prior oxidation (Fig. 1b). The Boc group was subsequently deprotected using the same protocol shown for **type 1** derivative, affording MWNTs-NH_3_^+^ (TGA mass loss of 15%, 529 μmol functional groups/g MWNTs). MWNTs-NH_3_^+^ (**type 2**) were dispersed in 5% dextrose at concentrations of 100 μg/mL and 1 mg/mL, affording **2-L** and **2-H**, respectively.

### Thermogravimetric Analysis

MWNT samples were loaded in platinum pans and the TGA analysis was performed using the TGA Q500 (TA instruments, USA). Initially the analysis was carried out isothermally in nitrogen atmosphere at 100°C for 20 min followed by a controlled increase in temperature at a rate of 10°C/min to reach a maximum of 1000°C at the end of the analysis.

### Length Analysis by Transmission Electron Microscopy (TEM)

Length distribution histograms were obtained by measuring nanotube length from transmission electron microscopy images (*N* = 100).

### Formulation of Cationic Liposomes and *f*-MWNTs-Liposome Hybrids

Liposomes, **L**, were prepared by lipid film hydration method followed by sonication. Briefly, DOTAP and cholesterol (2:1 molar ratio) were dissolved in chloroform:methanol (4:1 *v/v*), the organic solvent was evaporated in a rotary evaporator (Buchi, Switzerland) under vacuum at 40°C for 30 min and then flushed with a N_2_ stream to remove any residual traces of organic solvent. The dried lipid film was hydrated with 1 ml of 5% dextrose and multilamellar vesicles (MLV) were reduced in size by sonication for 2 min. Small unilamellar vesicles (SUV) were left for at least 2 h to anneal before being used or stored at 4°C under N_2_. For the preparation of hybrids **1-L**, **2-L** and **1-H**, **2-H**, the same protocol was used, but instead of hydrating the lipid film with 5% dextrose, solutions of 100 μg/ml and 1 mg/ml of *f*-MWNTs in 5% dextrose were used, affording hybrids with *f*-MWNT:lipid mass ratio of 5.3%:94.7 and 35.8%:64.2%, respectively. The final lipid concentration was 2 mM. Size reduction was achieved by bath sonication for 2 min.

### Electron Microscopy Imaging of the Hybrids

Low voltage STEM imaging was carried out on the FEI extreme high resolution Magellan 400 L SEM running at 20 kV fitted with a second generation FEI retractable STEM detector. This detector has multiple segments that allow capture of different signals simultaneously. The central part of the detector collects electrons at a normal angle producing a conventional bright field images. High angle annular dark field (HAADF) images are produced from scattered electrons that are collected on an annular dark field detector at higher angles; the intensity in HAADF images is directly related to the atomic number of the elements within the sample. Samples were sonicated and dispersed in 5% dextrose and placed dropwise onto a holey carbon copper support grid for STEM observation.

### Fluorescence Labelling of Cationic Liposomes and *f*-MWNTs-Liposome Hybrids

Fluorescently labelled liposomes and hybrids were prepared for fluorescence spectroscopy studies and *in vitro* cell uptake studies. Nile Red (lipid probe), emitting in the red region, is a hydrophobic fluorescent dye which was incorporated in the lipid bilayer of liposomes. Nile Red (NR) was added to the lipid mixture at a ratio of 1:300 dye:lipid molecules and formulations were prepared as described above. Formulations were kept protected from light.

### Physico-Chemical Characterization of Cationic Liposomes and *f*-MWNTs-Liposome Hybrids

Measurement of the hydrodynamic diameter and Zeta-potential of the prepared formulations were performed using dynamic light scattering (DLS) with a Nanosizer ZS series (Malvern Instruments, Southborough, MA). Disposable polystyrene cells and disposable plain folded capillary Zeta cells were used. Suspensions were diluted in deionized water and measurements were performed at 25°C. Electrophoretic mobility was used to calculate the ζ -potential using the Helmholtz-Smoluchowski equation. The hydrodynamic size was presented as the average value of 20 runs, with triplicate measurements within each run.

### Electrophoretic Mobility Shift Assay

The ability of *f*-MWNTs-liposome hybrids to complex siRNA was determined by GelRed displacement assay and agarose gel electrophoresis. Complexes were prepared by mixing 0.25 μg (25 μl) siNEG in 5% dextrose with an equal volume of DOTAP:chol liposomes or *f*-MWNTs-liposome hybrids at different Nitrogen (N)/ Phosphate (P) molar ratios. For example, 0.25 μg siRNA: 0.7011 μg DOTAP:Chol is equivalent to 1:1 N/P ratio. The positive charge contribution from *f*-MWNT was minimal so that the *f*-MWNT mass was not taking into account when calculating the N/P ratio. For comparison, 0.25 μg of siRNA was also mixed with *f*-MWNTs at equivalent ratios used in the hybrid. Complexes were incubated at room temperature for 30 min to allow complete formation. Naked non-coding siRNA (siNEG, 0.25 μg) was included for comparison. After staining with 6× Orange Loading Dye, the suspensions were loaded onto 1% (*w/v*) agarose containing 0.3× GelRed stain, followed by electrophoresis in 0.5× Tris-Borate-EDTA buffer (SigmaAldrich, UK) at 80 mV for 50 min. The gels were then visualized under UV light using ChemiDoc MP system (BioRad, UK).

### Nile Red Fluorescence Spectroscopy

NR fluorescence spectroscopy was used to study the interaction between the *f*-MWNTs and cationic liposomes in the formed hybrids. The fluorescence and emission spectra of Nile Red was measured using FLUOstar Omega (BMG labtech, UK) plate reader at excitation and emission wavelengths of 544 nm and 610–620 nm, respectively. A calibration curve using DOTAP:cholesterol liposome incorporating Nile Red (**L**) at a ratio of 1:300, dye:lipid molar ratio was established to confirm that fluorescence values were within the linear range of the instrument (data not shown). Different amounts of liposomes were used so that NR concentrations ranged from 0 to 1 μg/ml. Formulations were prepared as described above. This protocol was referred to as the “encapsulation protocol”. To exclude the quenching effect of *f*-MWNTs on the dye, control samples were prepared where NR was mixed with *f*-MWNTs in 5% dextrose (quenching control). Another control group was included in this experiment where *f*-MWNTs were mixed just before measurement with the preformed liposomes containing NR, at equivalent concentrations used in the encapsulation protocol. This protocol was referred to as the “mixing protocol”. For NR fluorescence measurements, 20 μl of each formulation (quenching control, encapsulation or mixing protocols) were diluted 5× to a final volume of 100 μl, yielding final lipid and NR concentrations of 0.6 mM and 0.625 μg/ml, respectively.

### *In Vitro* siRNA Stability in Serum

The extent of enzymatic degradation mediated by serum nucleases was analyzed by incubation of non-coding siRNA (siNEG) complexed with formulations **L** and hybrids **1-L**, **1-H**, **2-L**, and **2-H** with FBS. The complexes were prepared as described above, at N/P charge ratio of 4:1 as siRNA was fully complexed at this ratio as determined by the electrophoretic mobility shift assay. After complexation, FBS (5 μl) was added to the complexes at a final concentration of 50% (*v/v*) and samples were incubated at 37°C for 1 h or 4 h. To keep the same volume and incubation time as that used in the heparin competition assay, 5 μl of 5% dextrose were added to each sample and samples were further incubated at 37°C for 10 min. After incubation, the reaction was terminated by adding EDTA to a final concentration of 50 mM. Non-incubated naked siRNA (0.25 μg) or naked siRNA (0.25 μg) co-incubated with FBS (50% *v/v*) and EDTA (50 mM) were used as negative and positive controls, respectively. Samples were electrophoresed under the conditions described above.

### *In Vitro* siRNA Protection Against RNAse

The siRNA (siNEG) protection ability of formulations **L** and hybrids **1-L**, **1-H**, **2-L**, and **2-H** from RNAse was assessed. The complexes at N/P charge ratio of 4:1 were prepared as described above. After complexes were formed, RNAse (5 μl) was added to the complexes at a final concentration of 0.4 μg/ml and samples were incubated at 37°C for 10 min or 1 h. To keep the same volume and incubation time as that used in the heparin competition assay, 5 μl of 5% dextrose was added to each sample and samples were further incubated at 37°C for 10 min. After incubation, the reaction was terminated by adding EDTA to a final concentration of 50 mM. Non-incubated naked siRNA (0.25 μg) or naked siRNA (0.25 μg) co-incubated with RNAse (0.4 μg/ml) and EDTA (50 mM) were used as negative and positive controls, respectively. Samples were electrophoresed under the conditions described above.

### Heparin Competition Assay

A heparin competition assay was performed to dissociate siRNA (siNEG) complexes in order to examine the siRNA integrity following incubation either with FBS or with RNAse. Following incubation with either FBS or RNAse, heparin was added to each sample to a final concentration of 50 IU/ml and samples were further incubated at 37°C for 10 min. After incubation, the reaction was terminated by adding EDTA to a final concentration of 50 mM. Non-incubated naked siRNA (0.25 μg) or naked siRNA (0.25 μg) with RNAse (0.4 μg/ml), heparin (50 IU/ml) and EDTA (50 mM) were used as negative and positive controls, respectively. Samples were electrophoresed under the conditions described above.

### Cell Culture

Epithelial lung carcinoma cells (A549) were cultured in Ham’s F-12 supplemented with 10% FBS, 50 U/ml penicillin, 50 μg/ml streptomycin, 1% L-glutamine, at 37°C in 5% CO_2_. Luciferase expressing A549 cells (A549-Luc) were cultured in Ham’s F-12 supplemented with 10% FBS, 1% L-glutamine, at 37°C in 5% CO_2_. Cells were routinely grown in 75 cm^2^ canted-neck tissue culture flasks and passaged twice a week using 0.05% Trypsin/EDTA when reaching 80% confluency in order to maintain exponential growth.

### siRNA Uptake Studies in A549 Cells by Flow Cytometry

A549 cells were subcultured onto 24-well plates (5x10^4^cells/well) for 24 h prior transfection with liposome/siRNA or hybrid/siRNA complexes. Cells were incubated with complexes in serum-free and antibiotics-free medium for 4 h, after which serum was added to make the final concentration of serum to 10% (*v/v*). Cells were allowed to interact with the complexes for 24 h at 37°C in a humidified atmosphere (5% CO_2_) incubator. After incubation, cells were washed twice with PBS to remove unbound complexes, trypsinized, centrifuged at 800 g for 5 min, resuspended in PBS, and analyzed by flow cytometry. Whenever cells were not analyzed immediately, they were fixed in 4% (*v/v*) paraformaldehyde in PBS solution and stored at 4°C. Bivariate and univariate histograms were set using the BD FACSCalibur flow cytometer (Becton Dickinson, Franklin Lakes, NJ, USA) equipped with CellQuest Pro software (BD, USA). Ten thousand events were acquired in the gated cell population of interest. For NR-labelled liposomes or hybrids, cell uptake was assessed by NR fluorescence emission (FL-3 detector) and also by sideward scattering (SSC) as described previously (17, 18). Unlabeled non-coding siRNA (siNEG) was used in these experiments. For tracking siRNA internalization, ATTO 655-labelled siNEG was used in combination with non-fluorescently labelled **L** or the hybrids. ATTO 655-siNEG uptake was measured using FL-4 detector.

### Side Scatter Analysis (Complexity) in A549 Cells by Flow Cytometry

In the bivariate scatter histograms, the sideward scattering (SSC Lin, ordinate) *vs.* forward scattering histogram (FSC Lin, abscissa) was obtained. The forward scatter and side scatter detectors voltage had to be set in a way that both the negative control (a sample containing cells without any treatment) and the positive control cells (a sample containing cells that were transfected with DOTAP:chol/siRNA complexes) could be visualized inside the scatter dot plot. The population containing cells only was selected to include all the cells being studied and exclude any cell debris. In order not to include *f*-MWNTs in the cell population group, a suspension containing the highest concentration of *f*-MWNTs without any cells was run first to exclude any free CNT from the cell population. The univariate histograms of cell number (counts, ordinate) *vs*. either the sideward scattering intensity (SSC Lin, abscissa) or the fluorescence intensity (FL-3 Log for NR fluorescence or FL-4 Log for ATTO 655 fluorescence, abscissa) were plotted and gated for the cell population. The mean sideward scattering intensity values (SSC Lin) or the mean fluorescence intensity values (FL-3 and FL-4 Log) were recorded and used for comparison.

### Luciferase Expression in A549-Luc Cells

A549-Luc cells were seeded onto 96-well plates (1x10^4^ cells/well) with antibiotics-free F-12 medium 24 h prior transfection. The next day, siLuc or siNEG (10 μl) was complexed with an equal volume of formulations (**L** or **1-L**) in 5% dextrose at N/P charge ratio of 4:1. Following incubation at room temperature for 30 min, complexes were diluted in serum-free and antibiotics-free medium and added to the cells to achieve a final siRNA concentration of 80 nM. After 4 h, serum was added to reach 10% (*v/v*) final serum concentration. At 48 h post-transfection, cell lysate was prepared and luciferase expression was assayed with a Luciferase assay kit using a luminometer FLUOstar Omega (BMG labtech, UK) plate reader. Briefly, cells were washed with PBS before addition of 60 μl of lysis buffer. Two freeze-thaw cycles were performed to ensure complete cell lysis and cells were directly centrifuged in the 96-well plate for 30 min at high speed and 4°C. 20 μl of the supernatant containing proteins was mixed with 100 μl of luciferase assay reagent and then luminescence signals were recorded. 20 μl of the lysate was used to determine the protein concentration using a BCA protein assay kit, following manufacturer’s instructions. The Luciferase activity was calculated as RLU/mg protein. Values were normalized to siNEG transfected cells.

### Biological Activity of siPLK1 in A549 Cells With MTT Assay

In order to assess the functionality of siRNA delivered by **L** or **1-L**, a functional siRNA (siPLK1) was used and toxicity induced by PLK1-specific gene silencing was determined. A549 cells were seeded onto 96-well plates (1 × 10^4^ cells/well) with antibiotics-free F-12 medium 24 h prior transfection. The next day, siPLK1 or siNEG (10 μl) was complexed with an equal volume of the formulation (**L** or **1-L**) in 5% dextrose at N/P charge ratio of 4:1. Lipofectamine 2000 was used as a positive control for the transfection, whereas cells treated with 10% DMSO were used as a cytotoxic control for the MTT assay. Following incubation at room temperature for 30 min, complexes were diluted in serum-free and antibiotics-free medium and added to the cells, yielding a final siRNA concentration of 80 nM. After 4 h, serum was added to reach 10% (*v/v*) final serum concentration. At 48 h post transfection, MTT assay was performed. Cells were incubated with MTT solution at 0.5 mg/ml MTT final concentration for 3 h. Media was then removed and the formazan produced was dissolved in 200 μl DMSO and absorbance was read in a FLUOstar Omega (BMG labtech, UK) plate reader at 560 nm. Results were expressed as the percentage of cell viability (mean ± SD) and normalized to siNEG transfected grow of each carrier. The following equation was used: % cell viability = (A560 nm of siPLK1 transfected cells/ A560 nm of siNEG transfected cells) × 100.

### Apoptosis Measurement and Sub-G1 Analysis with Flow Cytometry

In order to assess the effect of combining PLK1 down-regulation with Dox toxicity using the different formulations on cell apoptosis (as a therapeutic endpoint), sub-G1 analysis was carried out by flow cytometry. MWNT has been reported to interfere with MTT assay so it was avoided here (19). A549 cells were seeded onto 24-well plates (5x10^4^ cells/well) for 24 h prior transfection. The next day, siPLK1 or siNEG was complexed with equal volume of formulation (**L** or **1-L**) in 5% dextrose at N/P charge ratio of 4:1. An equal volume of Dox was added to each complex and allowed to complex with MWNT for 30 min at room temperature, yielding a final Dox concentration of 1 μM. Cells were then incubated with the complexes (with or without Dox) in antibiotics-free medium for 48 h at 37°C in a humidified atmosphere (5% CO_2_) incubator. After incubation, both attached cells and those in suspension were harvested, washed in PBS and fixed with cold 70% (*v/v*) ethanol for at least 1 h, at 4°C. Following fixation, cells were centrifuged at 850 g for 5 min at 4°C to remove ethanol. The cells were then resuspended in PBS:citrate buffer (1:1 *v/v*) and incubated for 5 min at 37°C. After incubation, supernatant was removed by centrifugation at 850 g for 5 min at 22°C. The cells were then resuspended in PBS solution containing propidium iodide (50 μg/ml) and RNAse (100 μg/ml) and incubated in the dark for 30 min at 37°C. Cells were then analyzed by BD FACSCalibur flow cytometer. The percentage of events in the sub-G1 phase of the cell cycle histogram (10,000 events) was determined using CellQuest Pro software (BD, USA).

### Statistical Analysis

Data are presented as means ± standard deviation (SD). The statistical analysis was performed using one-way ANOVA followed by Tukey’s multiple comparison test (GraphPad Prism 5.0 software). Differences between groups were considered significant for *p < 0.05, highly significant for **p < 0.01 and extremely significant for ***p < 0.001.

## Results

### Physicochemical Characterization of *f*-MWNTs-Liposome Hybrids

Initially the length distribution of the *f*-MWNT was performed from TEM images (*n* = 100). The length of the pristine MWNT is provided by the supplier (0.5–2 μm). In case of oxidized MWNT (type 1), pristine MWNT is subjected to acid treatment step which results in shortening to 277.5 +/− 152.93 nm (20). In case of type 2 where no oxidation step was performed, the length was 455.5 +/− 185.42 nm. Cycloaddition reaction is not expected to reduce the length of MWNT (15), however, the centrifugation and filtration steps during functionalization leads to exclusion of longer tubes.

TGA is a technique widely used to characterize materials with different thermal stabilities. It was therefore used here to quantify the degree of chemical functionalization with organic functional groups (Fig. 1). In Fig. 1a, ox-MWNT-NH_3_^+^ (**type 1**, red line) showed a drop in weight loss at 150°C, due to the loss in the carboxylic groups introduced on CNT surface, and at 600°C due to graphitic-skeleton decomposition. ox-MWNT-NH_3_^+^ showed an increase in the weight loss of 13% at 600°C confirming the introduction of functional groups from the cycloaddition reaction. Pristine MWNTs were thermally stable up to 750°C (Fig. 1b, *black line*). MWNT-NH_3_^+^ (**type 2**) (Fig. 1b, *red line*) displayed an increase in the weight loss of 15% at 600°C also due to the introduction of functional groups from the cycloaddition reaction. The degree of chemical functionalization for ox-MWNT-NH_3_^+^ (**type 1**) and MWNT- NH_3_^+^ (**type 2**) was calculated as 193 μmol and 529 μmol functional groups/g MWNTs, respectively.

Physicochemical characterization of the four hybrids was also carried out to determine their feasibility as co-delivery systems. The composition, hydrodynamic diameter, polydispersity index (PDI) and ζ-potential of the different formulations tested are summarized in Table I. The hydrodynamic diameters of all formulations tested were, on average, less than 200 nm, with all *f*-MWNT-liposome hybrids showing greater sizes compared to cationic liposome (**L**). The reported hydrodynamic diameters of all hybrids were surprisingly smaller than the length of the corresponding MWNT (Fig. 1). An explanation for this could be the fact that the hydrodynamic diameter (Z-average) reflects a combined overall value and not the magnitude of just one dimension of the nanocarriers (21). Therefore, since the *f*-MWNTs are not spherical particles and their diameter is much smaller (20–30 nm) compared to their length, the hydrodynamic diameter of hybrids reflects a combined outcome of the two dimensions. Although DLS is not the best technique to measure the size of CNTs, we used this method as PDI values were indicative of the size distribution. PDI of hybrids **2-L** and **2-H**, formed with MWNT-NH_3_^+^, was greater than for those formed with ox-MWNT-NH_3_^+^ (hybrids **1-L** and **1-H**). This was expected as the CNTs that form hybrid type **2** are longer than the CNTs that form hybrid type **1**.

**Table I:** Physico-chemical characterization of the hybrids.

Formulation	CNT final concentration (μg/mL)	Formulation composition CNT:total lipid (*w/w*%)	Hydrodynamic diameter ± SD (nm)	Polydispersity index	**ζ**-potential ± SD (mV)
L	0	0 : 100	118.9 ± 1.83	0.256	48 ± 5.82
Hybrid 1-L	100	5.3 : 94.7	185.9 ± 1.12	0.319	52.4 ± 3.45
Hybrid 1-H	1000	35.8 : 64.2	167.1 ± 0.78	0.256	47.4 ± 0.80
Hybrid 2-L	100	5.3 : 94.7	166.4 ± 2.22	0.411	31.9 ± 1.80
Hybrid 2-H	1000	35.8 : 64.2	198.9 ± 4.15	0.354	51.8 ± 1.87

All formulations displayed positive ζ-potential values, ranging from 32 to 52 mV, comparable to that of cationic liposomes (48 mV).

### Morphological Studies

The morphology of the liposomes and hybrids was studied by low voltage STEM at 20 kV. This operating voltage allowed us to study the structure of these beam sensitive samples avoiding the knock-on damage occurring at higher operating voltages (>80 kV).

**Fig. 2:**
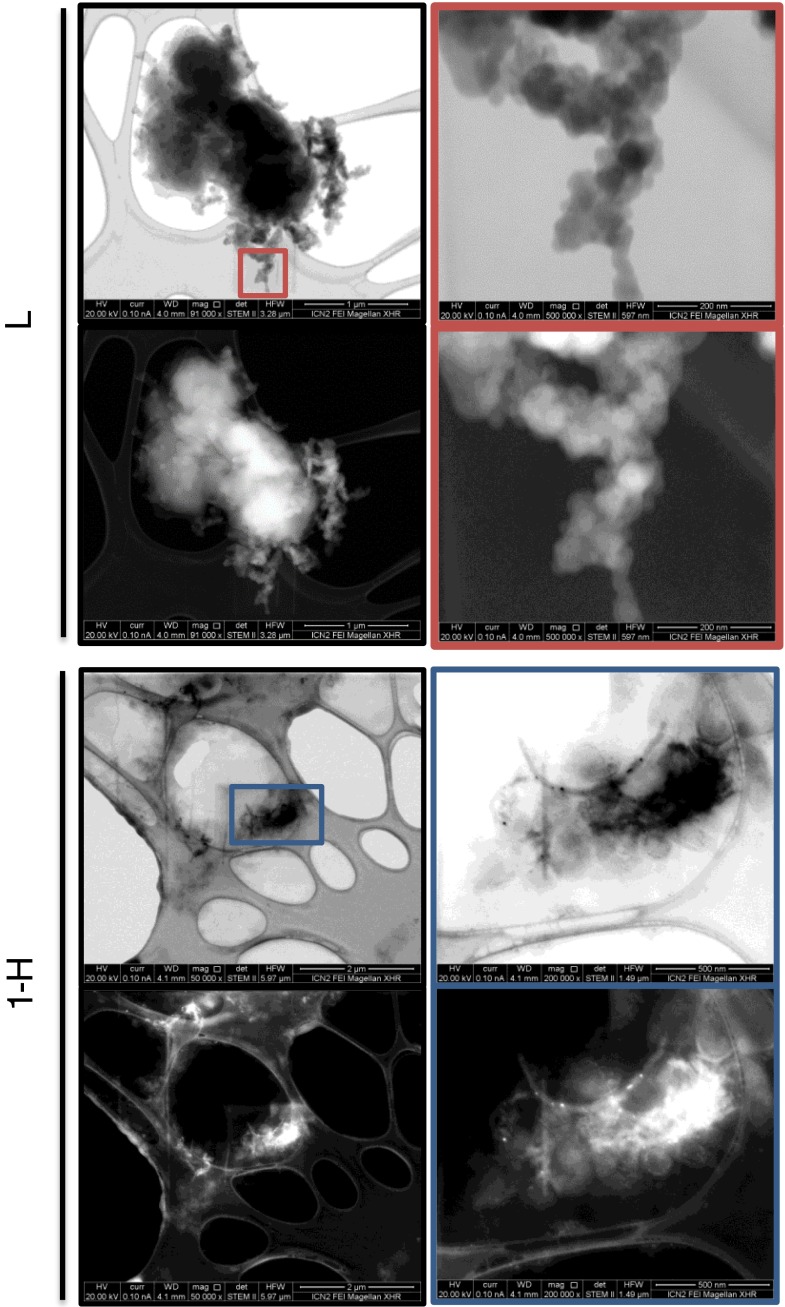
Morphological examination of **L** and **1-H**. Low voltage STEM images of liposomes (**L**) and the **1-H** hybrid.

Sample **L** showed round features of 60–200 nm in diameter in agreement with the hydrodynamic diameter of the cationic liposomes (Fig. 2). The cationic liposomes were found in micron-size agglomerates most probably formed upon solvent drying during the TEM grid preparation.

STEM images of sample **1H** (Fig. 2 and S1) showed a clear interaction of the cationic liposomes with MWNTs. Round features with faint contrast corresponding to the cationic liposomes were present close to the ox-MWNT-NH_3_^+^ and even embedding them.

AFM height images, in 2D and 3D for **L** and **1-H** are shown in Fig. S2. Cross-section analysis exhibited height of 5–10 nm for both samples indicating the collapse of the liposomes on the mica surface. Samples **1-H** showed more heterogeneous structures compared to liposome sample.

### siRNA Binding

**Fig. 3:**
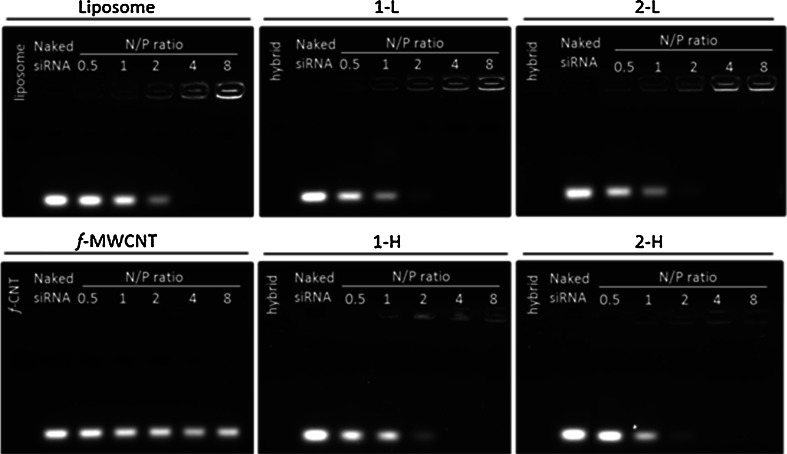
Electrophoretic mobility shift assay of the different formulations complexed with siRNA at various N/P ratios. Charge ratios are calculated for a fixed siRNA amount of 0.25 μg. Lane 1 – naked siRNA (0.25 μg), Lanes 2–6 – complexes at different N/P ratios.

The ability of the *f*-MWNT-liposome hybrids to complex nucleic acids and the optimum N/P ratio required for complexation were determined using an electrophoretic mobility assay across a range of N:P charge ratios from 0.5:1 to 8:1. The siRNA complexation profile of **1-L**, **1-H**, **2-L**, and **2-H** is shown in Fig. 3. DOTAP:chol liposomes (formulation **L**) and *f*-MWNTs were used as controls. DOTAP:chol liposomes and hybrids **1-L**, **1-H**, **2-L**, and **2-H** present a similar complexation profile, where full siRNA complexation was achieved at N/P ratio of 4:1, demonstrated by the absence of free siRNA band and concomitant increased fluorescence in the well. These results are in agreement with those obtained for ζ-potential measurements, where the overall surface charge of the hybrids remained unaltered upon hybridization with *f*-MWNTs. Therefore, it was expected that carriers ability to form complexes with siRNA would not be affected upon hybrid formation. This confirms the suitability of using the hybrids for siRNA delivery. *f*-MWNTs complexation profile in Fig. 3 represents the equivalent *f*-MWNTs amount present in the hybrid at the N/P ratios studied, which as expected afforded N/P ratios lower than those required to achieve complexation.

### Nile Red Fluorescence Spectroscopy

**Fig. 4:**
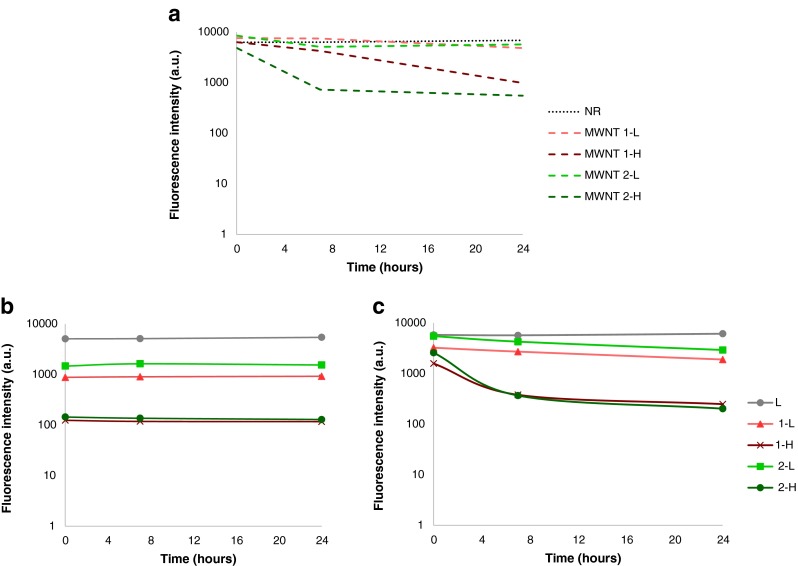
NR fluorescence spectroscopy for (**a**) NR mixed with *f*-MWNTs in 5% dextrose (quenching controls), (**b**) NR used in encapsulation protocol (the lipid film containing NR is hydrated with the *f*-MWNTs), (**c**) NR used in the mixing protocol (*f*-MWNTs were mixed with the pre-formed labelled liposomes) over time. NR fluorescence was detected at excitation and emission wavelengths of 544 nm and 610–620 nm, respectively. Fluorescence intensity values were measured at 0, 7 and 24 h. Results were expressed as means ± SD, *n* = 3.

Nile Red (NR) is a benzophenoxazone dye commonly used to study the interaction with lipid bilayers (22). It is hypothesized that when NR exists in non-polar environment, its fluorescence increases. A reduction in NR fluorescence in the hybrid form compared to liposome can be attributed to interactions between *f*-MWNT and liposome. The following groups were included in the study (Fig. 4): (i) NR mixed with *f*-MWNTs in 5% dextrose (quenching controls), (ii) NR incorporated into liposome during its formation then mixed with *f*-MWNT just before measurement (mixing control), (iii) NR incorporated into *f*-MWNT-liposome hybrid during its formation (encapsulation protocol). NR fluorescence intensities were monitored over a period of 24 h.

After 24 h incubation, *f*-MWNT **1-L** and **2-L** (Fig. 4a), showed a reduction of 22.8 and 9% in NR fluorescence intensity, respectively, compared to NR in 5% dextrose. When higher concentrations of *f*-MWNT **1-L** and **2-L** were used, mimicking their content in **1-H** and **2-H** hybrids, NR fluorescence reduced by 84.3 and 91.2%, respectively. In case of the hybrids prepared at a ratio of 5.3% (*w/w*) (Fig. 4b and c), when incubation time increased from 0 to 24 h, hybrid **1-L** and **2-L** showed reductions in NR fluorescence intensity by 82.7 and 70.3%, respectively, compared to **L**. This suggests that both types of *f*-MWNTs exhibited interactions with DOTAP:chol liposomes. NR quenching started to be seen at high *f*-MWNT concentrations (equivalent to 35.8% *w/w*) in both *f*-MWNTs and the hybrids (>84% reduction in fluorescence) (Fig. 4b and c). In summary, NR spectroscopy studies suggest that the lower NR fluorescence intensities obtained for the hybrids, of low f-MWNT content, compared to cationic liposomes could be a result of the interaction between *f*-MWNTs and liposomes. Conclusions on interactions could not be made at in 1-H and 2-H due to possibility of quenching.

### *f*-MWNT-Liposome Hybrids Stability Against Serum and RNAse

**Fig. 5:**
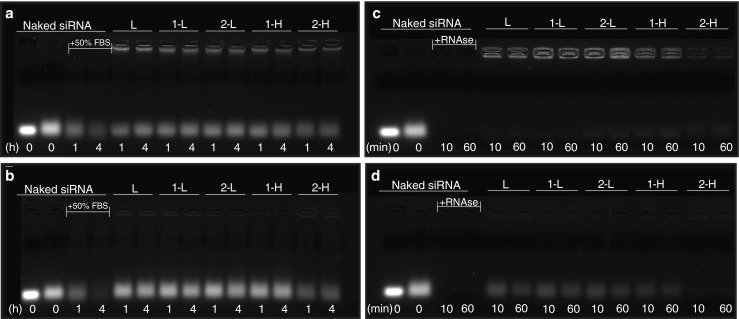
Agarose gel electrophoresis for complexes subjected to (**a**) serum; (**b**) serum followed by heparin competition assay of the different formulations complexed with siRNA at 4:1 N/P ratio; (**c**) RNAse; (**d**) RNAse followed by heparin competition assay of the different formulations complexed with siRNA at 4:1 N/P ratio. The complexes were prepared at a fixed siRNA amount of 0.25 μg. Complexes were incubated with 50% (*v/v*) FBS (1 h or 4 h) or RNAse (10 min or 60 min) at 37°C. For the heparin competition assay, complexes were further incubated for 10 min in the presence of 50 IU/mL heparin. 50 mM EDTA was used to inactivate serum proteins. In (**a**), (**b**) Lanes 1 and 2 (*far left*)– naked siRNA or naked siRNA + FBS + heparin + EDTA, respectively. In (**c**), (**d**) Lanes 1 and 2 (*far left*)– naked siRNA or naked siRNA + RNAse + heparin + EDTA, respectively.

The ability of the different *f*-MWNT-liposome hybrids to protect siRNA from nuclease mediated degradation was evaluated by challenging the siRNA complexes with serum. Naked siRNA, liposome/siRNA and hybrid/siRNA complexes were incubated at 37°C in 50% serum for 1 h or 4 h. As shown in Fig. 5a, after 4 h incubation with serum, naked siRNA was almost completely degraded, whereas both liposome and hybrids prolonged siRNA stability in serum at 4:1 N/P ratio. The heparin competition assay further confirmed that complex formation stabilized and protected siRNA in serum (Fig. 5b). The siRNA bands appeared at the same position as the control bands after dissociation of the siRNA/liposome or siRNA/hybrid with comparable intensities. Furthermore, incubation with serum up to 4 h did not seem to significantly affect the stability of these complexes.

The ability of complexes to protect siRNA against a ribonuclease (RNAse) was conducted by incubation for 10 min or 60 min at 37°C. As shown in Fig. 5c, naked siRNA was completely degraded after 10 min incubation with RNAse, whereas both liposome and hybrids prolonged siRNA stability in RNAse at 4:1 N/P ratio, demonstrated by the fluorescence in the wells. Following siRNA release mediated by heparin, hybrids **1-L** and **2-L** showed siRNA band intensities comparable to that of liposome (**L**) at the respective incubation times (Fig. 5d). In contrast, the extent of siRNA degradation by RNAse was higher for hybrids prepared with high concentration of *f*-MWNTs, in particular for **2-H** which was unable to protect siRNA from digestion at both time points. It is evident that **2-H** complexes are least stable compared to complexes formed with other hybrids thus, offering lowest siRNA protection against both serum and RNAse. This result is in agreement with NR fluorescence measurements where MWNT-NH_3_^+^ displayed a lower degree of interaction with cationic liposomes compared to ox-MWNT-NH_3_^+^. Interestingly, although all hybrids tested demonstrated similar siRNA complexation profiles, their siRNA protection capacities were different, indicating different stabilities, with the hybrids prepared with the lowest concentration of *f*-MWNTs presenting the most prominent protection ability which was comparable to that of **L**. As hybrid **2-H** did not appear to be very stable, hybrid type **2** was not included in the subsequent experiments. Furthermore, hybrids with lowest *f*-MWNT amount were preferred so next set of experiments was focused on hybrid **1-L**.

### ATTO 655-siRNA and Hybrid Cellular Uptake in A549 Cells

**Fig. 6:**
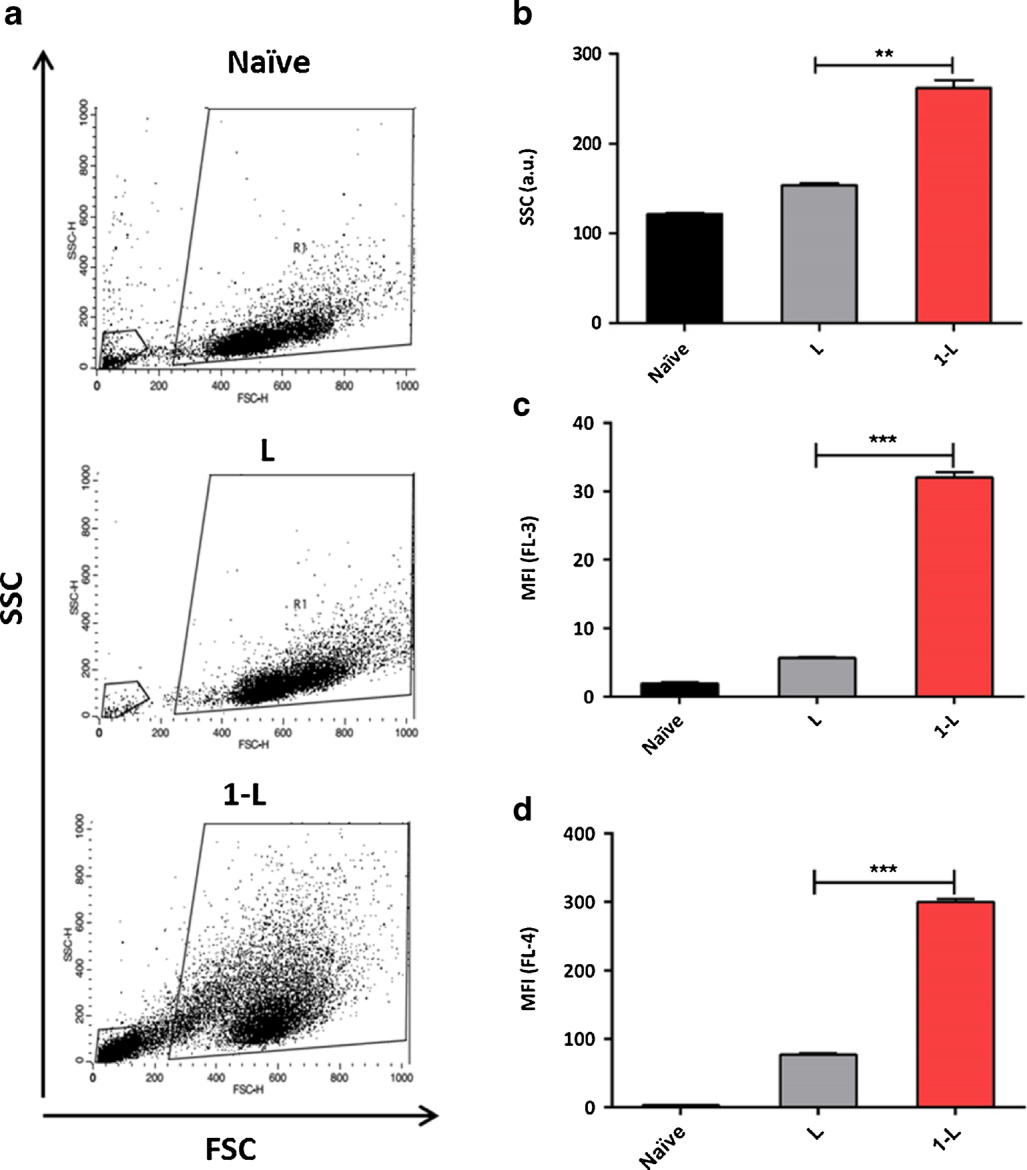
Analysis of cellular uptake of DOTAP:chol liposomes (**L**) and ox-MWNT-NH_3_^+^-DOTAP:chol hybrid (**1-L**) and complexed with siRNA in A549 cells using flow cytometry. (**a**) Bivariate scatter histograms of the sideward-scattering (SSC, Y-axis) *versus* forward-scattering (FSC, X-axis), (**b**) Mean sideward-scattering and (**c**) Mean Fluorescence Intensity of NR labelled **L** or **1-L** complexed with non-fluorescent siNEG. (**d**) Mean Fluorescence Intensity of ATTO 655-labelled siNEG complexed with unlabeled **L** or **1-L**. Flow cytometry analysis was performed after 24 h incubation with complexes. Experiments were done in triplicates and expressed as mean ± SD (*n* = 3).

To study the uptake of complexed hybrids in human A549 monolayers, DOTAP:chol/siRNA or **1-L**/siRNA complexes were incubated with cells for 24 h. The uptake of the carrier (**L** or **1-L**) was monitored by measuring Mean Fluorescence Intensity (MFI) of NR (FL-3) by flow cytometry. Uptake of ATTO 655 labelled-siNEG (emitting at λ_em_ = 684 nm)/(FL-4) allowed siRNA uptake quantification. Side scatter was used to monitor changes in cell complexity reflecting extent of *f*-MWNT uptake as we reported previously. Interestingly, when cells were allowed to interact with the hybrid complexes for 24 h, a significant increase in the sideward-scatter (SSC) by cells was observed compared to the naive (untreated cells) or cells treated with **L** complexes (Fig. 6a and b). In line with the SSC results, cells incubated with **1-L**/siNEG complexes displayed higher NR fluorescence than those incubated with **L**/siNEG complexes (Fig. 6c). Fluorescently labelled ATTO 655 siNEG was further employed to assess the siRNA delivery capacities of **1-L** compared to **L**, and to confirm if differences between the two delivery systems could actually be seen. Figure 6d represents MFI (FL-4) of cells incubated with ATTO 655 complexes after 24 h incubation with cells. A significantly higher siRNA uptake (~3 fold increase) was observed for **1-L** compared to **L**, consistent with the results obtained for NR labelled carriers.

### Functional Gene Silencing in A549 Cells

**Fig. 7:**
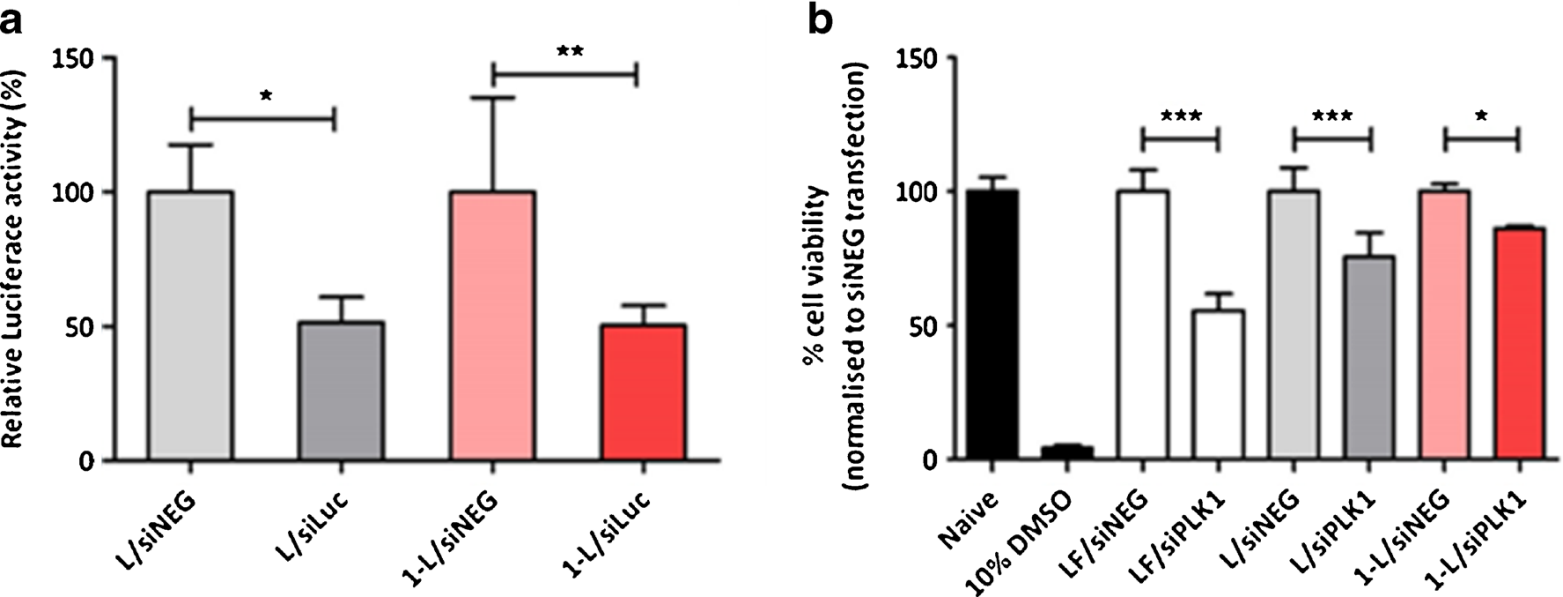
Analysis of gene silencing efficiency of DOTAP:chol liposomes (**L**) and ox-MWNT-NH_3_^+^-DOTAP:chol hybrid (**1-L**). (**a**) Relative luciferase activity in A549-Luc cells transfected with **L** or **1-L** complexed with non-coding siRNA (siNEG) or siRNA targeting Luciferase (siLuc). Cells were collected for luciferase and BCA assays 48 h post-transfection. (**b**) Cell viability (%) determined by MTT assay after 48 h transfection with **L** and **1-L** complexed with siNEG or siPLK1. Results were expressed as mean ± SD (*n* = 5).

The ability of *f*-MWNT-liposome hybrids to effectively silence a particular gene was studied using two experimental setups that evaluated gene silencing activity. First, A549-Luc cells were transfected with siLuc (siRNA that silence the non-functional Luciferase gene) using **L** or **1-L** (Fig. 7a). A comparable level of Luciferase silencing was achieved at 48 h by the two systems of 48.4% ± 11.1 (**L**) and 49.6% ± 9.6 (**1-L**). Consistent with the results obtained for Luciferase silencing, **L** and **1-L** induced comparable levels of PLK1 silencing which resulted in -20% cell kill at 48 h post-transfection (Fig. 7b).

### siPLK1 and Doxorubicin Co-Delivery to A549 Cells

**Fig. 8:**
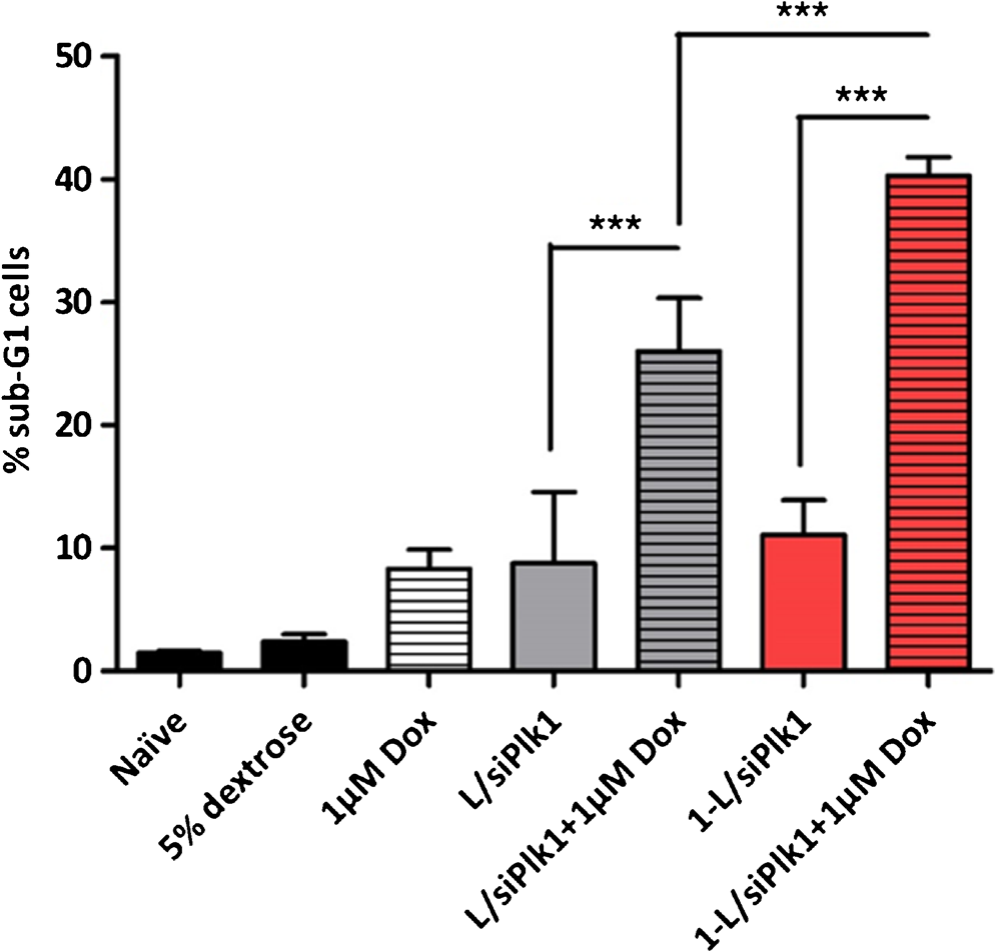
Growth inhibition and induction of apoptosis in A549 cells by the co-delivery of siPLK1 and Dox with DOTAP:chol liposomes (**L**) or ox-MWNT-NH_3_^+^-DOTAP:chol hybrid (**1-L**). Sub-G1 analysis using propidium iodide staining and flow cytometry was performed 48 h after transfection. Cells treated with 1 μM of Dox with or without PLK1 silencing with **L** or **1-L** vectors. siNEG transfected cells were used as negative controls and the toxicity of each system was normalized to its siNEG control. Results were expressed as mean ± SD (*n* = 5).

To assess the feasibility of the new hybrids as co-delivery systems, we next carried out sub-G1 analysis to evaluate cellular apoptosis induced by PLK1 depletion and Dox toxicity using **L** and **1-L** (Fig. 8). A549 cells were incubated with liposomal system **L** and **1-L** complexed with siPLK1 (with or without 1 μM Dox) and sub-G1 analysis was performed using flow cytometry after 48 h of incubation. Cells incubated with 1 μM Dox or transfected with siPLK1 as a single agent were used as positive controls. Interestingly, when Dox and siPLK1 were co-delivered to A549 cells, a synergistic effect was achieved with both carriers **L** and **1-L**. More importantly, the synergism between siPLK1 and Dox dual therapy was significantly more pronounced when the two agents were delivered by **1-L** (40.3% ± 1.5 apoptotic cells) compared to system **L** (26.0% ± 4.3 apoptotic cells).

## Discussion

Several studies reported that the co-delivery of a chemotherapeutic agent and siRNA significantly enhances the cytotoxicity to cancer cells and has great potential to overcome the drug resistance. This approach also reduces the side effects of chemotherapy as lower dosages of the cytotoxic drug are needed to achieve similar therapeutic effect. However, reports on delivering siRNA simultaneously with a conventional anticancer drug to cancer cells for enhanced chemotherapy efficacy have been scarce, due to the lack of efficient co-delivery methods (23, 24). Due to PLK1 altered expression in a wide range of cancers and to its roles in cancer progression, it was the chosen target to siRNA therapy. The cationic nature of liposomes is essential to efficiently complex the negatively charged siRNA *via* electrostatic interactions and for interaction with the negatively charged cell membrane.

Recently, Karchemski *et al.* reported the use of CNTs-liposomes conjugate in an attempt of combining the efficient cell uptake of CNTs with the well-known high drug loading capacity of liposomes, which may result in the prevention of potential adverse systemic side effects as lower amount of CNTs are required while enhanced cellular uptake can still be obtained (25). In another study, Miyako *et al*. successfully developed self-assembled CNT-liposome supramolecular nanotrains, which function as intelligent molecular-transport systems. Utilizing the photothermal property of CNTs and the temperature-responsive property of liposomes, controlled released was achieved by illumination of laser causing the destruction of liposome and therefore the release of its cargo (26). By taking advantage of the highly efficient cellular uptake and siRNA delivery of cationic liposomes and the high drug loading capacity of CNTs and their ability to shuttle cytotoxic drugs into cells, we aimed at formulating *f*-MWNTs-cationic liposome hybrids for the simultaneous delivery of siRNA and an anticancer drug to cancer cells.

We demonstrated that siPLK1 delivery by **1-L** led to significant silencing of the target protein and that a synergistic effect was achieved when Dox and siPLK1 were co-delivered to A549 cells *in vitro*. All hybrids displayed ζ –potential values comparable to that of cationic liposomes (Table I) which indicates that *f*-MWNTs incorporation did not affect the overall cationic surface charge of the final hybrids formed and so that they could still be used to complex siRNA. The minimal N/P ratio for full complexation of siRNA and *f*-MWNT-cationic liposome hybrids was found to be 4:1 (Fig. 3), similar to that obtained for cationic liposome and consistent with previous studies.

The fact that the overall charge of liposome and complexation ability was not affected upon hybrid formation suggests that *f*-MWNTs were not associated with the liposome surface, so hydrophobic interactions were proposed. To address this question, NR fluorescence spectroscopy was carried out to investigate the nature of interaction between *f*-MWNTs and cationic liposomes. If the interaction of the *f*-MWNTs is to occur with the lipid bilayer, this should cause a localized disruption in its environment thus altering its microenvironment polarity and therefore changing the spectral characteristics of NR. We hypothesize that upon interaction of liposomes with *f*-MWNTs, the bilayer becomes leakier and hence more polar, leading to a decrease in NR fluorescence intensity. The fact that NR fluorescence was lower for all hybrids, of low *f*-MWNT content, compared to **L** could indicate the presence of interaction between the *f*-MWNTs and cationic liposomes. The quenching of dye fluorescence in the presence of CNTs was seen at high *f*-MWNT concentrations agreeing with a previously reported study (27).

Instability poses a major problem in siRNA applications because it degrades in serum within only a few minutes. We tested the ability of the different *f*-MWNT-liposome hybrids to protect siRNA from nuclease mediated degradation by challenging the siRNA complexes with serum and RNAse. Agarose gel electrophoresis results (Fig. 5) indicated that hybrids **1-L**, **1-H**, and **2-L** confer similar siRNA protection abilities to cationic liposomes under test conditions. On the other hand, siRNA complexes formed with hybrid **2-H** seem to be less stable, proved by the lower intensity of siRNA bands compared to all other bands. A possible explanation for the lowest stability of hybrid **2-H** and hence the reduced protection ability may be due to the poor dispersibility of MWNT-NH_3_^+^ in aqueous solution, potentially leading to particle aggregation and consequent disruption of liposome structure. This result is consistent with NR fluorescence data where MWNT-NH_3_^+^ displayed a lower degree of interaction with cationic liposomes compared to ox- MWNT-NH_3_^+^. Taking into account the low stability of hybrid type **2** and the fact that lower amounts of CNTs are preferred, next experiments were carried out with hybrid **1-L**.

To study the uptake of carriers **L** and **1-L** and of siRNA in A549 cells, NR and ATTO 655 labeled-siNEG fluorescence were monitored, respectively. SSC results showed increased cell complexity for **1-L** compared to **L** (Fig. 6a and b), suggesting enhanced cell uptake with **1-L**. Furthermore, cells incubated with **1-L** complexes showed increased NR and ATTO 655 fluorescence compared to **L** (Fig. 6c and d). The enhanced siRNA uptake with this hybrid could be due to enhanced cellular internalization (compared to **L**) (Fig. 6b). Overall, the enhanced cellular uptake of the *f*-MWNT-cationic liposome hybrid compared to cationic liposomes alone revealed a promising relevance of this system for the delivery of genetic material.

Although cell uptake was improved when siRNA was delivered by **1-L** compared to **L**, both Luciferase and PLK1 silencing experiments revealed comparable gene silencing activity of **1-L** and **L** (Fig. 7a and b).

**1-L** was then utilized as a co-delivery system for the delivery of siRNA and a cytotoxic drug *in vitro*. The rationale behind combining PLK1 silencing with chemotherapy was that combined therapies may prolong G2/M phase arrest, rendering cells more prone to apoptosis instead of mitotic slippage (28). Sub-G1 analysis was performed in A549 cells to evaluate cellular apoptosis induced by PLK1 depletion and Dox toxicity using **L** and **1-L**. It is possible that Dox interacts with MWNTs *via* Π-Π stacking of Dox aromatic chromophore groups and MWNTs backbone, as we previously reported (29).

When 1 μM Dox was co-delivered with siPLK1, both carriers achieved a synergistic effect which was significantly more pronounced for **1-L** compared to **L**. These results correlate well with the enhanced cellular uptake of this system and put forward hybrid **1-L** as a promising system for the co-delivery of siRNA and a cytotoxic drug and encourage further *f*-MWNT-liposome characterization and *in vivo* studies.

## Conclusion

Post-transcriptional gene silencing by double-stranded siRNA constitutes an attractive approach to cancer therapy. However, in order to become a therapeutic modality, novel delivery systems which allow direct siRNA translocation into the cytoplasm of the target cells together with an efficient gene silencing ability are of the most importance. In the present study, *f*-MWNT-cationic liposome hybrids were formulated and tested for their siRNA binding ability, stability, cellular uptake, *in vitro* gene silencing efficiency, induction of apoptosis and siPLK1 and Dox co-delivery efficiency. DOTAP:chol liposomes were used as an established model for siRNA delivery and used for comparison. All hybrids investigated here were capable of fully complexing with siRNA at N/P charge ratio of 4:1. Formulations where the hydrophobic dye Nile Red was embedded in the lipid bilayer were prepared and interaction between *f*-MWNTs and cationic liposomes was confirmed by fluorescence spectroscopy. Hybrids prepared with the lower concentrations of *f*-MWNTs showed serum and RNAse stabilities similar to those obtained for DOTAP:chol liposomes and therefore the subsequent studies were carried out with these systems. Cellular uptake studies in A549-Luc cells demonstrated the enhanced uptake ability of hybrid **1-L**. Moreover, gene silencing experiments with siLuc and siPLK1 revealed that **1-L** was able to efficiently silence Luciferase and PLK1 genes to levels comparable to cationic liposomes. In addition, flow cytometry analysis of sub-G1 population showed that these systems were capable of inducing cell apoptosis following PLK1 depletion by siPLK1. When Dox was co-delivered with siPLK1 by these systems, a synergistic effect was achieved with both liposomal system **L** and hybrid **1-L**, with the latter yielding a significantly higher number of apoptotic cells. The results presented here encourage further testing of **1-L** system as a potential vehicle for the co-delivery of siRNA and cytotoxic drugs. Structural modifications that favor the *in vivo* use (*e.g.,* the incorporation of a fusogenic lipid such as DOPE or PEGylation of the liposome surface) should be considered.

## Electronic Supplementary Material

ESM 1 (DOCX 2698 kb)

## ABBREVIATIONS

1-H: ox-MWNTs-NH_3_^+^-liposome hybrid (*f*-MWNT:total lipid mass ratio 35.8%:64.2%)
1-L: ox-MWNTs-NH_3_^+^-liposome hybrid (*f*-MWNT:total lipid mass ratio 5.3%:94.7%)
2-H: MWNTs-NH_3_^+^-liposome hybrid (*f*-MWNT:total lipid mass ratio 35.8%:64.2%)
2-L: MWNTs-NH_3_^+^-liposome hybrid (*f*-MWNT:total lipid mass ratio 5.3%:94.7%)
chol: Cholesterol
CNTs: Carbon nanotubes
DLSl: Dynamic light scattering
DOTAP: 1,2-dioleoyl-3-trimethylammonium-propane
Dox: Doxorubicin
*f*-CNTs: Functionalized carbon nanotubes
*f*-MWNTs: Functionalized multi-walled carbon nanotubes
L: Liposome
Luc: Luciferase
MFI: Mean Fluorescence Intensity
MTT: 3-(4,5-dimethylthiazol-2-yl)-2,5-diphenyltetrazolium bromide
MWNT-NH_3_^+^: Ammonium-functionalized multi-walled carbon nanotubes
NR: Nile Red
ox-MWNT-NH_3_^+^: Ammonium-functionalized oxidized multi-walled carbon nanotubes
PDI: Polydispersity index
PLK1: Polo-like kinase 1
RISC: RNA-induced silencing complex
RNAse: Ribonuclease
siNEG: non-coding siRNA
siRNA: Small interfering RNA
SSC: Sideward scattering
