# Supplementary material for: Cationic Liposome- Multi-Walled Carbon Nanotubes Hybrids for Dual siPLK1 and Doxorubicin Delivery In Vitro
Source: Pharm Res. 2015 Jun 18;32(10):3293–308. doi: 10.1007/s11095-015-1707-1 (PMC4577551; doi:10.1007/s11095-015-1707-1)
Supplement: Supplementary file 1 — (DOCX 2698 kb) [file 11095_2015_1707_MOESM1_ESM.docx]

**Cationic liposome- multi-walled carbon nanotubes hybrids for dual siPLK1 and doxorubicin delivery *in vitro***

*Sara Pereira, Jin Lee, Noelia Rubio, Hatem A. F. M. Hassan, Izzat Bin Mohamed Suffian, Julie T.W. Wang, Rebecca Klippstein, Belén Ballesteros, Wafa’ T. Al-Jamal*, and Khuloud T. Al-Jamal**

Miss S. Pereira, Miss J. Lee, Dr N. Rubio, Dr J. T.-W. Wang, Mr H.A.F.M. Hassan Mr I.B.M. Suffian, Dr R. Klippstein and Dr K. T. Al-Jamal

Institute of Pharmaceutical Science

King's College London

Franklin-Wilkins Building

150 Stamford Street

London SE1 9NH (UK)

E-mail: [khuloud.al-jamal@kcl.ac.uk](http:///h)

Dr *Belén Ballesteros*

ICN2 - Institut de Catala de Nanociencia i Nanotecnologia

Campus UAB

08193 Bellaterra, Barcelona (Spain)

Miss S. Pereira and Dr W. T. Al-Jamal

School of Pharmacy

University of East Anglia

Norwich Research Park
Norwich NR4 7TJ (UK)

E-mail: [wafa.al-jamal@uea.ac.uk](file:///C:\Users\Sara%20Pereira\AppData\Local\Temp\Temp1_PHAM-D-14-00535.zip\wafa.al-jamal@uea.ac.uk)

__________________________________________________

* To whom correspondence should be addressed.

E-mail: [khuloud.al-jamal@kcl.ac.uk](mailto:khuloud.al-jamal@kcl.ac.uk); [wafa.al-jamal@uea.ac.uk](mailto:wafa.al-jamal@uea.ac.uk)

**SUPPLEMENTARY INFORMATION**

# **Atomic force microscopy (AFM)**

# 100 µl of poly-l-lysine solution (PL) (Sigma-Aldrich, Deisenhofen, Germany) was deposited on freshly cleaved mica (11 mm × 11 mm × 0.15 mm). After incubation for 30 s, the mica was rinsed with 4 ml of millipore purged water, the water was soaked up, and finally, the mica was dried in a nitrogen stream. The surface topography of **L** or **1-H** samples deposited on a PL-coated mica surface was studied with AFM using tapping mode. Aqueous dispersions were used without dilution, deposited on the PL-coated mica for 5 minutes at room temperature Excess sample was removed and the mica was dried under a gentle flow of compressed nitrogen (or a dust-free compressed air spray). The sampling was achieved by oscillating the tapping probe to hit the sample surface, which allowed short-time interactions with minimal shear force applied on the surface. The oscillation of reflected laser spot signal from the probe cantilever (general purpose tips cat: NSC15/AL, Mikromasch, USA) was collected with ScanAsyst® Dimension Icon® AFM (Bruker, UK). The AFM images visualization and analysis were achieved using WSxM v5.0 Developed 6.2 software (Nanotec Electronica S.L., Madrid, Spain).

**Figure S1**- Morphological examination of **1-H** by low voltage STEM images.

**Figure S2**- AFM height images in 2D and 3D. **L** and **1-H** showed a cross-section height of 5 ~ 10 nm, indicating collapse of the liposomes on the mica surface. Samples **1-H** showed more heterogeneous structures compared to liposome sample.
